# Longitudinal MRI template of the baboon brain from birth to adolescence

**DOI:** 10.1162/IMAG.a.1316

**Published:** 2026-07-30

**Authors:** Katherine L. Bryant, Arnaud Le Troter, David Meunier, Yannick Becker, Scott A. Love, Siham Bouziane, Kep Kee Loh, Julien Sein, Luc Renaud, Olivier Coulon, Adrien Meguerditchian

**Affiliations:** Institute for Language, Communication, and the Brain, Aix-Marseille Univ, CNRS, Marseille, France; Centre de Recherche en Psychologie et Neurosciences, Aix-Marseille Univ, CNRS, UMR 7077, Marseille, France; Institut de Neurosciences de la Timone, Aix-Marseille Univ, CNRS, UMR 7289, Marseille, France; Department of Neuropsychology, Max Planck Institute for Human Cognitive and Brain Sciences, Leipzig, Germany; Lise Meitner Research Group Cognitive Neurogenetics, Max Planck Institute for Human Cognitive and Brain Sciences, Leipzig, Germany; INRAE, CNRS, Université de Tours, PRC, 37380, Nouzilly, France; Department of Psychology, National University of Singapore, Singapore; Station de Primatologie, CNRS-CELPHEDIA UAR846, Rousset, France

**Keywords:** structural imaging, comparative, primate, evolution, resource

## Abstract

The baboon (*Papio*) is an invaluable resource within nonhuman primate research, having the advantage of being a cercopithecoid (Old World monkey) with one of the largest brains among non-hominid primates. In order to facilitate comparative developmental neuroscience research, we present the BABACOOL (BAby Brain Atlas COnstruction for Optimized Labeled segmentation) approach for creating multi-modal developmental atlases, which we used to produce BaBa21, a population-based longitudinal developmental baboon template. BaBa21 is a spatio-temporal template that consists of structural (T1- and T2-weighted) images and tissue probability maps from a population of 21 baboons (*Papio anubis*) scanned at 4 timepoints beginning from about 2 weeks after birth and continuing to sexual maturity (5 years). Further, this study offers a fully automatic method for generating a template at any intermediate age for future age-specific group studies. This resource is made available to provide a normalization target for baboon data across the lifespan, including intermediate timepoints, and moreover facilitate neuroimaging research in baboons, comparative research with humans and nonhuman primate species for which developmental templates are available (e.g., macaques).

## 1 Introduction

As the field of non-human primate (NHP) neuroimaging expands, recent efforts have focused on sharing resources and standardizing approaches to accelerate progress (Friedrich et al., 2021; Milham et al., 2018). As new primate species are analyzed in a comparative neuroimaging framework, this not only advances our understanding of brain organization, but also sheds light on the features that make human brains unique (Mars et al., 2014), with clear clinical implications (Rogers Flattery et al., 2020). Adding a longitudinal dimension to magnetic resonance (MR) templates offers further opportunities to understand the development of brain structures over time at both individual and population levels.

Population-based species-specific neuroimaging templates offer a normalization target that permits workers to register their primate acquisitions to a standard and thus effectively analyze their data in a validated anatomical space, and further, segment tissue types using established probabilistic priors. A previous baboon (*Papio anubis*) T1w template, Haiko89 (Love et al., 2016) is based on 89 individual adults. Here, we offer a developmental template generated from a cohort of 21 individual baboons (*P. anubis*) scanned at four time points, starting about 2 weeks after birth through adolescence.

Developmental templates, especially those based on longitudinal data from the same cohort, are labor- and resource-intensive, but offer a plethora of advantages to developmental neuroscience researchers (Cusack et al., 2018). In pediatric studies, investigators are confronted with rapid brain changes due to postnatal maturation, necessitating age-specific templates. In recent years, developmental templates using longitudinal data have been generated for humans (e.g., Richards et al., 2016) and macaques (e.g., Tan et al., 2025; Zhong et al., 2022). In addition to the utility of these templates for researchers with scans of neonates or juveniles, developmental templates in other NHP species open the door to investigations into the relationship between ontogeny and phylogeny.

The most recent common ancestor of baboons (*Papio*) diverged from that of humans approximately 32 million years ago (Pozzi et al., 2014), when crown Catarrhines (Old World anthropoids) split into the Hominoidea and Cercopithecoidea. Along with members of the *Mandrillus* genus, members of *Papio* possess the largest adult non-hominid brains among Primates in terms of absolute size (Heldstab et al., 2018; Isler & van Schaik, 2012; van Woerden et al., 2012); with *P. anubis* averaging approximately 160 cm^3^ (Meguerditchian et al., 2021), over one-and-a-half times larger than rhesus macaques. Life history characteristics include a terrestrial lifestyle, omnivory, gestation of a single offspring, and a relatively long gestational period (6 months) during which the brain matures dramatically (Leigh, 2004). Sexual maturity is reached by males and females between the 5th year and 6th year; however, males take several additional years to reach full maturity in terms of size (Altmann et al., 2010).

These life history characteristics, phylogenetic closeness to humans, and large brain size all make baboons valuable for understanding human brain evolution as well as human-specific neuropathologies. Specifically, baboons are a useful model for understanding the evolution of language (Fagot et al., 2019; Meguerditchian, 2022) and cerebral hemispheric specialization (Becker & Meguerditchian, 2022) social systems (Fischer et al., 2019), and culture (Claidière et al., 2018). With regard to neuropathology, they are an established model for epilepsy (Szabó et al., 2012) and stroke (Kwiecien et al., 2014), and are more recently being used in studies of Parkinson’s disease (Arotcarena et al., 2020) and age-related cognitive decline more broadly (Lizarraga et al., 2020).

Apart from the macaque (Zhong et al., 2022) and marmoset (Sawiak et al., 2018), few developmental templates exist for NHPs. Here, we offer a population-based developmental multimodal (T1w and T2w) baboon brain template with accompanying tissue probability segmentations in a common stereotactic space.

## 2 Methods

### 2.1 Subjects

All animal procedures were approved by the Ethical committee of neurosciences C2EA-71 (INT Marseille; APAFIS#13553-201802151547729 v4) and were conducted at the Station de Primatologie (SdP; Rousset-Sur-Arc, France) and the Centre IRM (INT Marseille, Centre Européen en Imagerie Médicale) under agreement C130877 for conducting experiments on vertebrate animals. All methods were performed in accordance with the relevant French law, CNRS guidelines, and the European Union regulations (Directive 2010/63/EU).

All subjects were free from developmental problems, neurological antecedents, and brain abnormalities. The facility housing baboons at SdP consists of outdoor enclosures with wood and metal ethologically approved structures for enrichment. Feedings are four times a day and consist of seeds, monkey pellets, and fresh fruits and vegetables; water is available *ad libitum*. The total number of scanned subjects was 31, of which 21 had a complete set of scans of high quality and reached the last third longitudinal time point. These 21 were used in the construction of the template. Due to segmentation failures in two cases and poor image quality in one subject, 20 valid scans were available at both ses-0 and ses-1. As a result, the number of usable images slightly varied across developmental stages (see Table 1).

**Table 1.** Age distribution of subjects across longitudinal timepoints.

Time point	Age group	N (# males)	Mean age (days)	Mean age (months)
0	Newborn	20 (12)	29.3 ± 17.2	0.9 ± 0.6
1	Weaned	20 (12)	275.8 ± 35.1	9.2 ± 1.2
2	Juvenile	21 (12)	745.2 ± 21.7	24.8 ± 0.7
3	Sexually Mature	21 (12)	1712.8 ± 28.1	57.1 ± 0.9

### 2.2 Protocol

Mothers and their infants were isolated from their social group the evening before the scan at SdP for a medical check. Mother and newborn were transported together the following day to the MRI center (Centre IRM, Institut Neurosciences de la Timone, Marseille, France). Upon arrival at the MRI center, the mother of the focal subject was sedated with an intramuscular injection of ketamine (3 mg/kg) and medetomidine (30 μg/kg). Neonates were then separated from the mother and sedated using 4–7% sevoflurane mixed with O_2_. Subjects over 5 months old were sedated with 2–3% sevoflurane. Tracheal intubation was performed using a Sengstaken-Blakemore tube to assure artificial respiration in case of emergency. Tracheal intubation was performed for steady controlled ventilation using an anesthetic ventilator (Fabius MRI, Drager, Germany). Anesthesia was maintained with 3% sevoflurane via a calibrated vaporizer with a mixture of air (0.75 L/min and O_2_ 0.1 L/min). O_2_ saturation and cardiac rate were monitored throughout acquisitions with a 2 spO_2_ device placed on the lower lip and a respiratory belt equipped with a mechanical sensor. End-tidal carbon dioxide, halogen gas level, and volume/pressure was monitored and used to adjust ventilation rate (0.2–0.3 Hz) and end-tidal volume via an MRI respirator.

Each subject was placed in a ventral decubitus position in the scanner with the head secured by foam positioners, cushions, and velcro strips to maintain position and reduce potential motion. Noise protection was attached around the ears. A forced-air warming blanket and hot water bottles were used to maintain homeostatic temperature of the focal subject during all procedures. Ophthalmic ointment (vitamin A) was applied to protect eyes from dryness. At the end of the MRI session, subjects were monitored as they recovered from anesthesia. Neonates and young baboons were returned to the mother in the cage after her recovery from anesthesia. Mother and infant were monitored to make sure the mother responded to the infant’s needs (breast feeding, etc.). Focal subjects and mothers were returned to the SdP and rejoined their social group the same or the following day.

### 2.3 Imaging parameters

From September 2017 to March 2020, *in vivo* imaging was performed using a 3T clinical MRI scanner (MAGNETOM Prisma, Siemens, Erlangen, Germany) equipped with 80 mT/m gradients (XR 80/200 gradient system with slew rate 200 T/m/s), a 2-channel B1 transmit array (TimTX TrueForm). For timepoint 0, 1, and 2, two 11 cm receive-only loop coils with one under the head and another one around the face were used, and for timepoint 3, a 20-channel human head/neck coil was used. T1-weighted (T1w) images were acquired using a 3D-MPRAGE (Mugler & Brookeman, 1990) sequence (0.4 mm isotropic, FOV = 103 x 103 x 102.4 mm, matrix = 256 × 256, slices per slab = 256, sagittal orientation, readout direction of inferior to superior, phase oversampling = 10%, averages = 3, TR = 2500 ms, TE = 3.01 ms, TI = 900 ms, flip-angle = 8°, bandwidth = 300 Hz/pixel, no fat suppression). T2-weighted (T2w) images were acquired using the Sampling Perfection with Application optimized Contrast using different angle Evolutions (SPACE) sequence (Mugler et al., 2000), with the following parameters: 0.4 mm isotropic (0.5 for timepoint 3), FOV = 154 x 115.5 x 102.4 mm, matrix = 384 × 288, slice per slab = 256, sagittal orientation, readout direction I to S, phase oversampling = 0%, TR = 3200 ms, TE = 393 ms, bandwidth = 566 Hz/pixel, no fat suppression, echo train length = 790 ms, and pre-scan normalization. The total acquisition time was 65 minutes for the structural imaging (35 minutes for T1w and 30 minutes for T2w) out of a 2-hour session which included additional sequences (BOLD, DWI, and QSM).

### 2.4 Multimodal template creation

The BaBa21 template was constructed in a space compatible with the Haiko89 adult baboon template (Love et al., 2016): oriented in AC-PC, with a similar field of view, and a similar native spatial resolution—(0.4–0.5 mm)^3^ compared to Haiko89’s (0.4 mm)^3^. This longitudinal version comprised acquisitions of the same individuals at four points in time, temporally distributed from newborn (2–4 weeks; “timepoint 0”), weaned (9 months; “timepoint 1”), juvenile (2 years; “timepoint 2”), and sexually mature (5 years; session “timepoint 3”). Twenty-one individuals of the entire dataset were selected for inclusion in the template (n = 21; 12 males; Table 1).

T1w volumes were initially co-registered to the Haiko89 T1w template (Love et al., 2016), downsampled to (0.6 mm)^3^, using the antsRegistration function with the following parameters: rigid-only transformation, gradient step = 0.1, shrink factor (8 x 4 x 2), smoothing factor (3 x 2 x 1), and maximum iterations (1000 x 500 x 250). Using these transformations, all volumes of the dataset were rigidly resliced into the Haiko89 space and left/right flipped in order to generate an approximately symmetrical template. More details are given in the sections below.

**Fig. 1.**
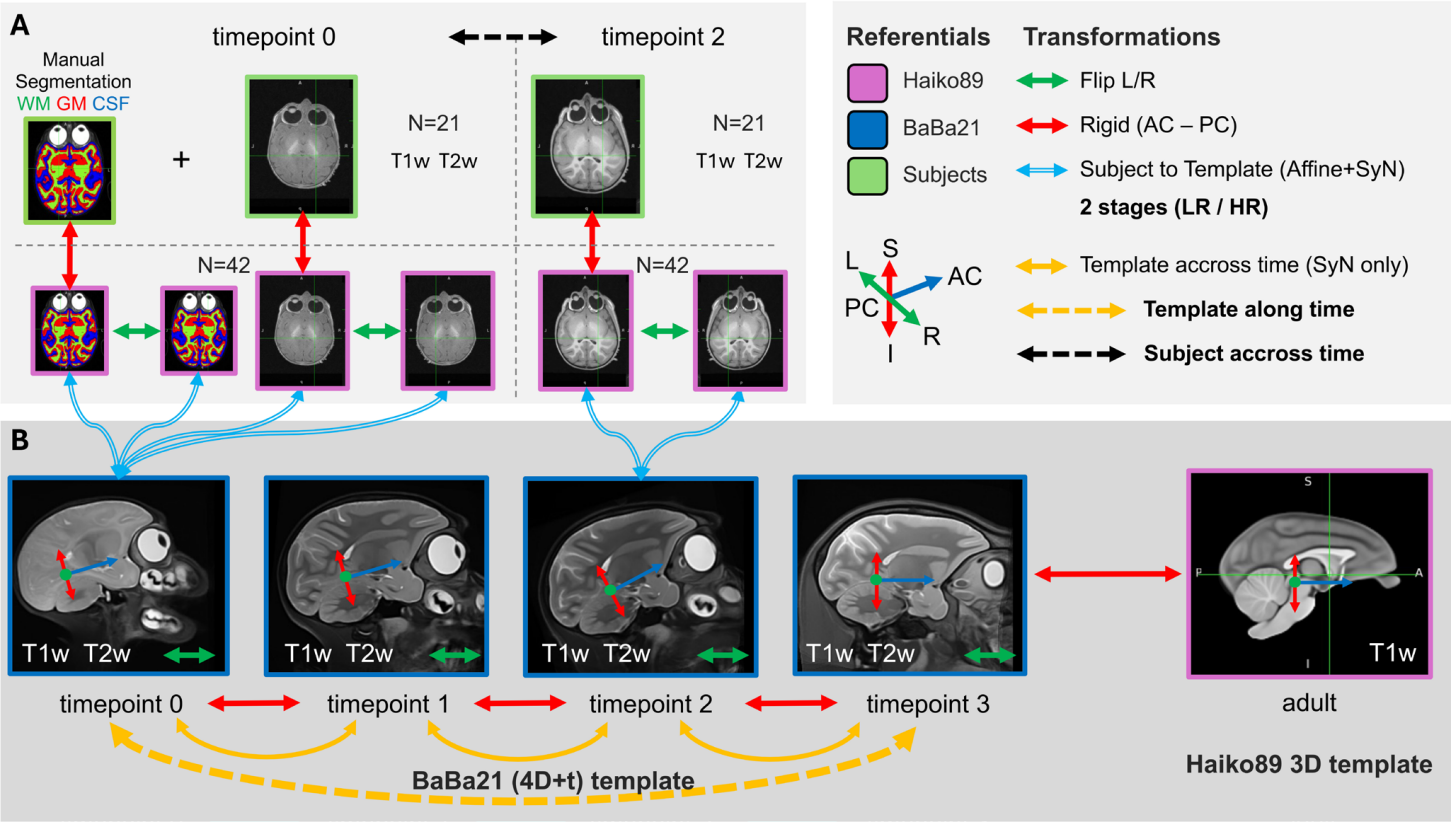
Schematic of the BaBa21 template generation pipeline. First, population-averaged templates are generated for each timepoint (A); second, templates are normalized across timepoints via rigid AC-PC re-alignment, non-linear registration, and (4D+t) interpolation in one single field of deformation (B).

An iterative SyGN pipeline in ANTs (Avants et al., 2011), using the antsMultivariateTemplateConstruction2.sh script, was used in two steps to generate the multimodal template, pooling T1w and T2w contrasts (Fig. 1A). In the first step, T1w and T2w images were downsampled to an isotropic voxel size of (0.6 μm)^3^, cropped to a matrix size of 180^3^ voxels in Haiko89 space, with maximum iterations set to 30 x 20 x 10. In the second step, the preprocessed T1w and T2w images were resampled to an isotropic voxel size (0.4 mm)^3^, cropped to a matrix size of 270^3^ in Haiko89 space, with maximum iterations set to 50 x 30 x 15. For both steps, the process consisted of four iterations with the following parameters: shrink factor 4 x 2 x 1, smoothing factor 2 x 1 x 0, and cross-correlation for pairwise registration. The output of the first step was interpolated to 0.4 mm isotropic resolution, serving as the input for the second step. Laplacian sharpening (Wang, 2007) was applied to the T1w and T2w templates at each iteration for both steps. For a detailed list of steps, see template_construction.md in the BaBaCool github repository.

For timepoint 0, the low T1w contrast near the cortical ribbon poorly resolved the GM/WM interface; thus, for this timepoint only, manually segmented WM binary masks were integrated as a third contrast into template creation. SyGN registration was constrained by assigning weights (0.5 for WM mask, 0.5 for T1w, 1.0 for T2w) during the maximization of the global similarity metric, permitting a clearer appearance of the cortical ribbon in the template space. A visual comparison of the template before and after the addition of the WM mask prior demonstrates the improvement in contrast at the GM/WM interface (Supplementary Fig. S1).

### 2.5 Alignment across timepoints

Templates from the four longitudinal sessions were rigidly aligned with AC-PC anatomical landmarks. The timepoint-(i) session was chosen as the target for the realigned timepoint-(i-1) session, with timepoint 3 being the initial target and Haiko89 the initial source. In the timepoint 3 session, three orthogonal planes based on the AC and PC landmarks were manually identified: the axial plane (xy, determined by [AC, PC] and [L, R] vectors), the inter-hemispheric sagittal plane (yz, determined by [AC, PC] and [S, I] vectors), and the coronal plane (xz, orthogonal to the others, determined by [L, R] and [S, I] vectors including the PC point; red, green, and blue arrows in Fig. 1).

For each consecutive timepoint (timepoint 2, timepoint 1, timepoint 0), a multi-modal registration was determined based on T1w and T2w contrasts in two steps. First, a multi-stage transformation (rigid+affine+SyN) was performed to calculate precisely the location of the PC (the origin of the 3 planes) and the 3 planes. In a second step, the resulting backpropagated landmarks were used to estimate a single rigid transformation that can bring the PC and the three planes into the native space of the timepoints (timepoint 2, timepoint 1, timepoint 0) while preserving orthogonality of the 3 planes. The transformations were used to propagate segmentation masks from Haiko89 to timepoint 3, timepoint 3 to timepoint 2, timepoint 2 to timepoint 1, and timepoint 1 to timepoint 0. Next, the three planes were segmented into the four timepoints. The previous longitudinal registration scheme was repeated, using only rigid transformation based on a cross-correlation similarity metric estimated solely on the propagated segmentation masks. After this step, BaBa21 templates for the first three timepoints were reoriented across the four timepoints. Finally, the multi-modal templates were left/right flipped and averaged to generate a temporary symmetrical template, which was used as the final target for a last rigid registration step. This step produced an exact symmetrical template by averaging both the co-registered template and its flipped version.

### 2.6 Tissue probability maps and binary brain mask generation

Tissue probability maps (TPM) for white matter (WM), gray matter (GM), and cerebrospinal fluid (CSF) were generated in the following manner. After automatic segmentation with Macapype (macatools.github.io/macapype), WM segmentations were manually corrected in subject spaces by an expert in anatomy (SB) for timepoint 0, timepoint 1, and timepoint 2. Timepoint 3 did not require manual corrections due to the image quality being similar to adult scans. Timepoint 0 required more substantive corrections in all brain regions (especially the cerebellum) than other timepoints, which was achieved by utilizing T2w contrasts, which offered better visibility of the GM/WM interface than T1w images alone (Supplementary Fig. S1). All final segmentations of GM, WM, and CSF tissues were deformed by applying all geometric transformations—rigid to Haiko89 and rigid + affine + nonlinear to BaBa21 spaces—and averaged to build the new tissue probability maps.

Binary thresholding (values greater than 0.2) was applied to the TPMs for GM and CSF. The resulting binary masks were combined, followed by a hole-filling operation (26-connectivity). Morphological erosion using a 3D kernel (3 x 3 x 3 voxels) was then performed to generate the final binary brain mask (BM). The TPMs for GM and CSF were generated by masking them with the BM. The WM TPM was subsequently regenerated from the binary BM by subtracting the corrected TPM values for GM and CSF to ensure that the sum of the GM, WM, and CSF probability values in each voxel equals 1.

### 2.7 Histogram-based normalization for T1w/T2w intensity transformation

The Laplacian filter applied during the SyGN process, after averaging for each iteration, enhances the tissue contrasts and improves the resolution of anatomical details. This permits improved segmentation and registration between subject and template but also contributes to increasing the differences in T1w and T2w value intensity ranges across timepoints. To reduce these variations, T1w and T2w maps were normalized using the multiclass histogram-based approach (Sun et al., 2015), using the following equation:

$$\begin{array}{l} T1w_{norm}\;\left(x,y,z\right)=a.T1w\;\left(x,y,z\right)+b\\ T2w_{norm}\;\left(x,y,z\right)=c.T2w\;\left(x,y,z\right)+d \end{array}$$ (1)

Where (a, b) and (c, d) are respectively variables of a system of linear Equations (2) and (3), respectively for normalization of T1w and T2w maps as described below:

$$\begin{array}{l} 70=a.T1w\;{\left(WM\right)}_{90th}+b\\ 30=a.T1w\;{\left(GM\right)}_{10th}+b \end{array}$$ (2)

$$\begin{array}{l} 30=c.\;T2w\;{\left(WM\right)}_{10th}+d\\ 70=c.\;T2w\;{\left(GM\right)}_{90th}+d \end{array}$$ (3)

Note that Laplacian sharpening is optional for this step of template generation. Also note that prior to this intensity normalization, an N4 bias correction is applied on templates in order to remove any intensity bias effects. It is also possible to correct for the B1 bias in all images prior to template construction. Tests showed that the resulting template is equivalent to the method presented here. Nevertheless, the pipeline provided at https://github.com/arnaudletroter/BABACOOL/blob/main/postprocessing/bias_correction.md offers the possibility to use both strategies (bias correction before or after template construction).

### 2.8 Generation of intermediate timepoint templates

To interpolate intermediate timepoint templates, a multimodal SyN-only registration was applied to the volumes that were previously rigidly aligned (see Section 2.5). Once the multi-modal SyN-only registration, based on T1w and T2w contrasts, converged across two successive timepoints, warp and inverse warp deformation fields were generated. Then, each BaBa21 template modality (T1w, T2w, TPM) was deformed over time along the series of diffeomorphisms connecting them, creating a linear interpolation along the SyN geodesic path (Avants et al., 2008; Fu et al., 2017) between timepoint 0 and timepoint 1. Next, each BaBa21 template modality (T1w, T2w, TPM) was deformed over time along the series of diffeomorphisms connecting them. Intermediate volumes were generated by applying fractional SyN transformations along the geodesic path. For each image pair, forward and inverse diffeomorphic transformations were estimated using the Symmetric Normalization (SyN) algorithm implemented in ANTs (Avants et al., 2008). Let $\phi_{1}$ denote the forward transformation from image I to image J, and $\phi_{2}$ the corresponding inverse transformation. The warped images $\phi_{1}\left(I\right)$ and $\phi_{2}\left(J\right)$ were combined to generate intermediate volumes according to

$$K\left(t\right)=\;\frac{t\phi_{1}\left(I\right)+\left(1-t\right)\;\phi_{2}\left(J\right)}{2},$$

where t∈[0,1] weights the relative contribution of the forward-warped source image and the inverse-warped target image.

To further investigate WM consistency across timepoints and qualitatively assess the behavior of the interpolation function, WM TPMs were thresholded at 0.5 to generate binary masks, from which surfaces were subsequently extracted for each original and interpolated timepoint. This surface-based representation enables a direct visual examination of the spatial continuity induced by the interpolation, allowing us to evaluate whether intermediate shapes evolve smoothly and remain anatomically consistent with the original timepoints.

To examine the accuracy of the intermediate timepoint templates, we compared the BaBa21 model (broken down into 11 timepoints between ses-0 and ses-1) to a single subject (Ozone) which had not been included in our template generation due to the fact that it was scanned at a timepoint intermediate to ses-0 and ses-1. This comparison allows us to determine whether the intermediate interpolated anatomy reasonably fits the measurements for Ozone at the actual intermediate timepoint. We quantified this fit by estimating the amount of volumetric changes between a given timepoint and ses-1 of Ozone.

Ozone was scanned at 165 days (between ses-0 and ses-1 timepoints for BaBa21) and 265 days (within the range of ses-1 for BaBa21). To avoid confusion with the Baba21 timepoints, we will call these two time points Ozone_ses-0_ and Ozone_ses-1_ in the text below. These scans were compared to 11 models from BaBa21: ses-0 (29 days), ses-1 (275 days), and 9 models of interpolated intermediate timepoints between those two timepoints.

Ozone’s session 1 scan was manually segmented with the same methodology that was applied to the BaBa21 subjects. WM and GM maps were then non-linearly registered (using rigid+affine+SyN transforms) to Ozone_ses-0_. The WM volume change between Ozone_ses-0_ and Ozone_ses-1_ was then estimated (Supplementary Fig. S2).

For each BaBa21 time point, we compared the volume of WM between a given time point and the Baba21/ses-1. We can then assess which of the BaBa21 timepoints has the most similar WM volumetric change to the change observed in Ozone between Ozone_ses-0_ and Ozone_ses-1_. Volume changes are then compared to the reference (Ozone_ses-0_ to Ozone_ses-1_) changes by computing a volume ratio similarity as follows: WM volumes are computed as the sum of values in the WM TPM of each image, and the similarity is:

$$VRS\left(t\right)=\;\frac{V_{WM}\left(Ozone_{ses-1}\right)}{V_{WM}\left(Ozone_{ses-0}\right)}-\;\frac{V_{WM}\left(BaBa21_{ses-1}\right)}{V_{WM}\left(BaBa21_{ses-t}\right)}$$

## 3 Results

### 3.1 Templates and tissue probability maps

**Fig. 2.**
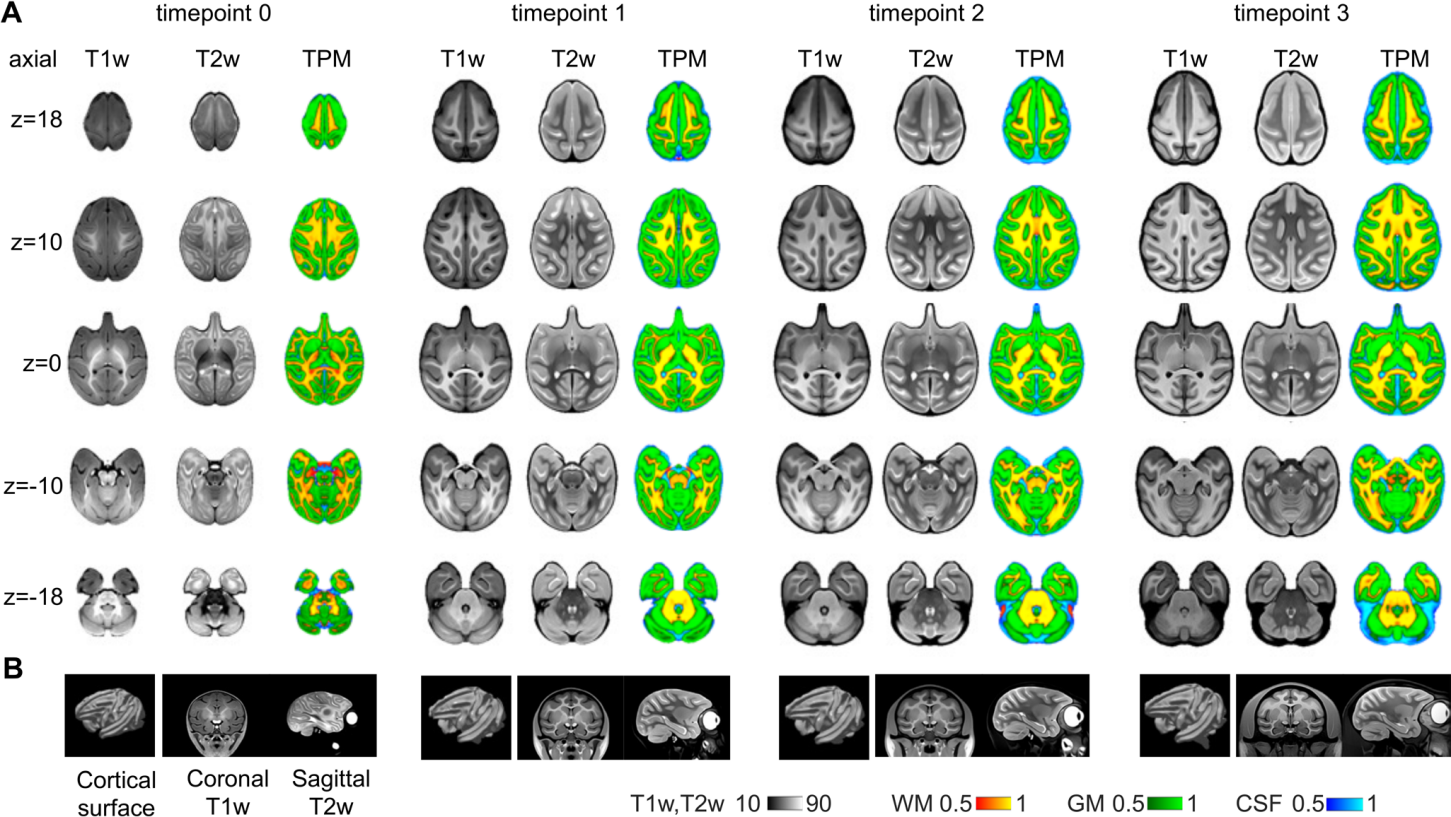
BaBa21 (4D+t) template with (A) representative axial slices from T1w, T2w, and TPM volumes by column at 5 positions along the z axis (z = -18, z = -10, z = 0, z = 10, z = 18) and (B) 3D cortical surface, coronal T1w and sagittal T2w views.

Multimodal population-averaged templates integrating T1w and T2w images were created for all four timepoints as described in Section 2 (Fig. 2). As described in Section 2.6, the classification of tissue types into gray matter, white matter, and cerebrospinal fluid was generated as part of the segmentation within the BABACOOL pipeline (Fig. 2; see Supplementary Fig. S3 for an individual subject’s TPM). TPMs were precise enough to resolve even narrow WM/GM boundaries in the extreme capsule at timepoint 0 (Supplementary Fig. S1).

**Fig. 3.**
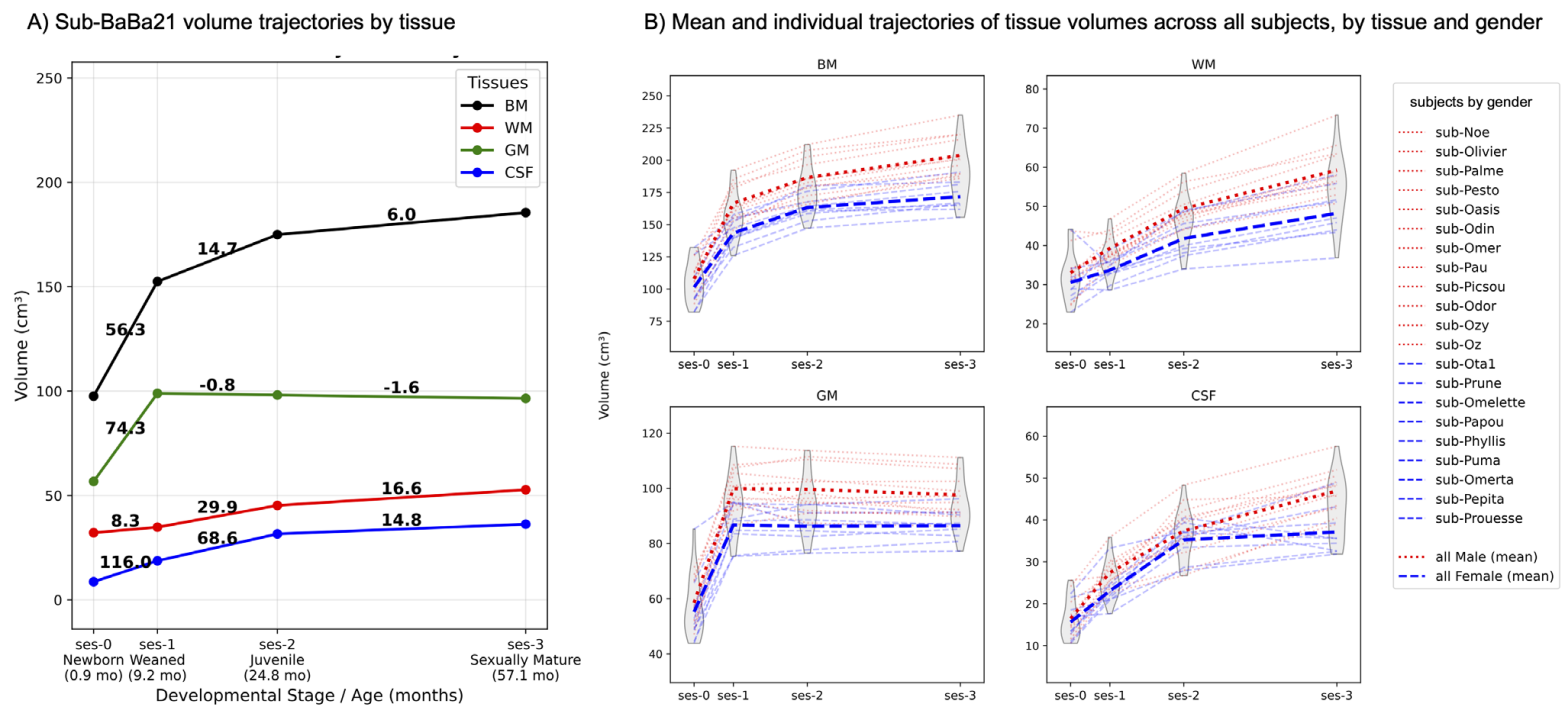
Tissue volume trajectories across timepoints at the template level (A) together with the same trajectories per subject and split by sex (B).

TPMs were optimized using a histogram-based normalization approach (described in Section 2.7). Before normalization, inter-timepoint variability in T1w and T2w values were apparent (Supplementary Fig. S4A), while after, tissue intensity values were centered within 30–70% of the normalized maximum intensity (Supplementary Fig. S4B). In order to properly describe the demographics of our group, we provide in Figure 3 the tissue volume trajectories across timepoints at the template level (Fig. 3A) together with the same trajectories per subject and split by gender (Fig. 3B).

### 3.2 Intermediate timepoint templates

**Fig. 4.**
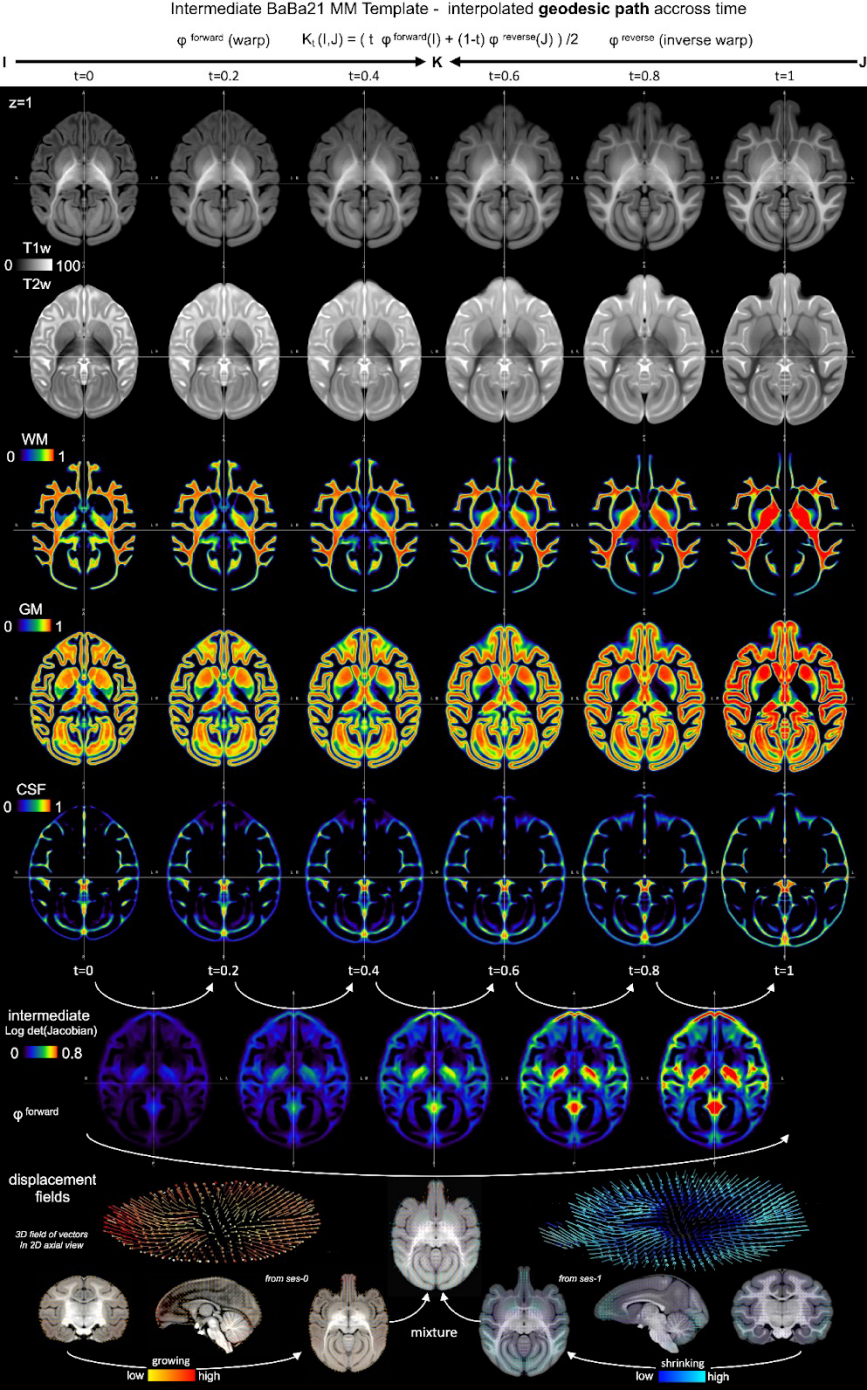
Intermediate BaBa21 multimodal template between timepoint 0 and timepoint 1 using interpolated geodesic path across time.

Intermediate timepoints for all template modalities (T1w, T2w, TPM) were linearly interpolated between timepoint 0 and timepoint 1 (Fig. 4). Normalized T1w, T2w, and TPM volumes are represented in rows (on a single axial slice at z = 0), for a set of intermediate interpolated timepoints K at time t= [0,0.2,0.4,0.6,0.8,1], shown in columns (Fig. 4). The last two rows show intermediate deformation fields, weighted by the interpolation factor t, are visualized through the logarithm of Jacobian determinant and the global deformation field from timepoint 0 to timepoint 1 as a vector field (Fig. 4) and the full forward deformation fields between successive timepoints (Supplementary Fig. S7).

Comparing the interpolated timepoints between ses-0 and ses-1 with values from an individual subject scanned between those timepoints (175 days) showed that the interpolated estimate was close to the measured value (Supplementary Fig. S5).

**Fig. 5.**
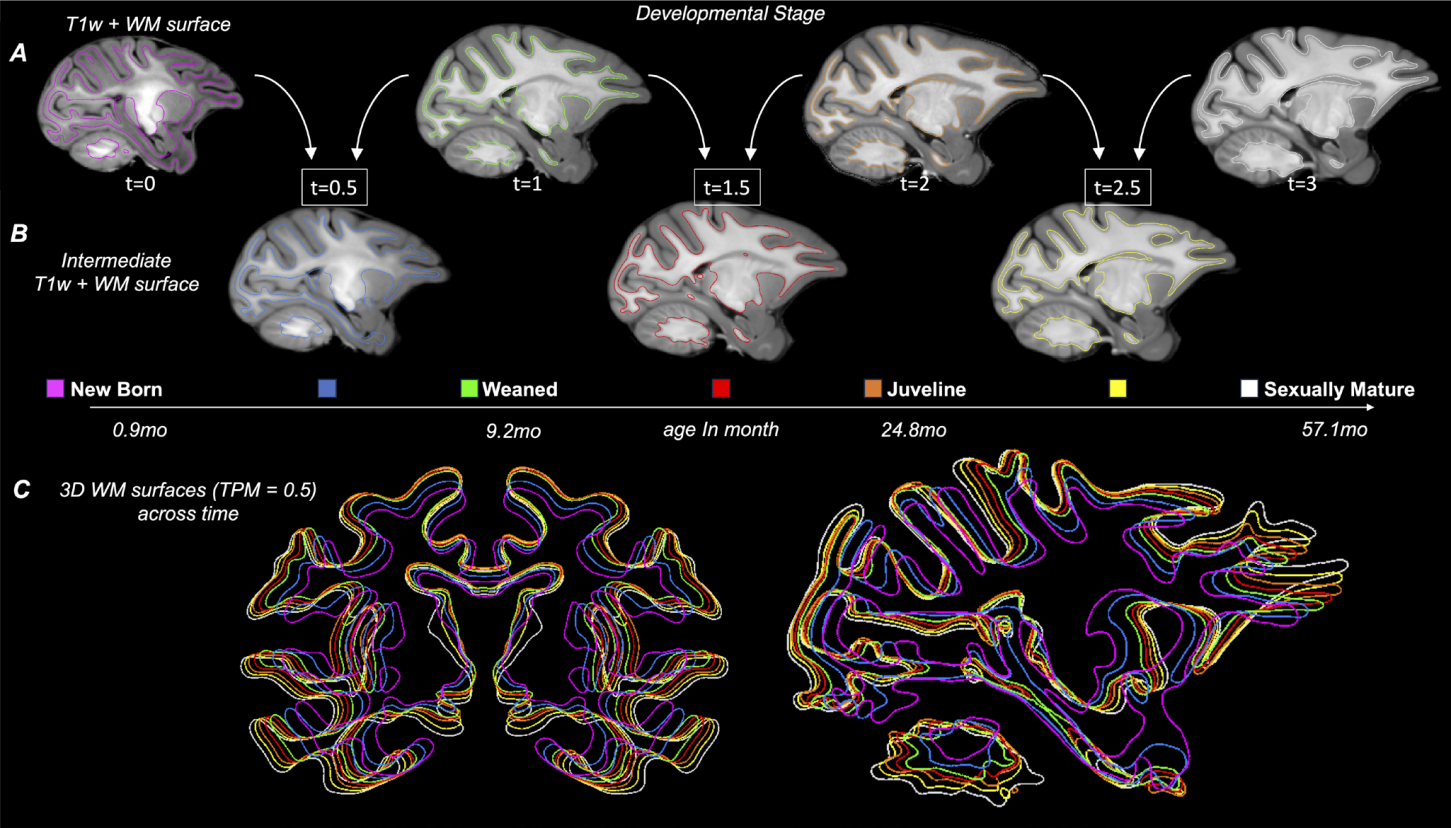
(A) Sagittal views for the four original T1w/WM BaBa21 templates (timepoints t = 0, t = 1, t = 2, t = 3) and (B) Three intermediate template generated by the geodesic path across time (timepoints t = 0.5, t = 1.5, t = 2.5), (C) Coronal and Sagittal views of 3D White matter (WM) surfaces extracted from thresholded tissue probability maps (TPMs) (threshold = 0.5, row C) across time.

The resulting WM surfaces derived from thresholded TPMs are shown in Figure 5. Sagittal views of the four original templates (t = 0, 1, 2, 3) together with the intermediate templates generated along the geodesic trajectory (t = 0.5, 1.5, 2.5) demonstrate a continuous temporal progression of anatomical shape. The WM surfaces exhibit smooth spatial transitions between consecutive timepoints, without visible discontinuities or abrupt geometric changes, supporting the stability of the interpolation process. Overall, these qualitative observations confirm the anatomical plausibility and temporal coherence of the interpolated templates.

## 4 Discussion

### 4.1 Summary

BABA21 is a longitudinal developmental baboon brain template, complementing and building on a previously released adult baboon brain template, Haiko89 (Love et al., 2016), which uniquely integrates T1w and T2w contrasts. This template includes 4 timepoints from neonate to sexual maturity, providing a longitudinal averaged template designed to measure tissue evolution over development. This is an essential resource for brain development studies that relies on quantitative morphometric analyses performed directly from tissue segmentation, and further, facilitates the multi-species comparisons necessary for investigating the relationship between brain development and evolution.

Like Haiko89, BaBa21 is a symmetric template, which facilitates analysis of lateralization through voxel-based morphometric approaches. In humans, the link between tissue volume differences between hemispheres and function is well-established, with left-lateralized white matter volumes observed in human language areas (Pujol et al., 2002), and correlations between language lateralization and relative gray matter volumes (Josse et al., 2009). In recent years, the baboon has been explored as a model for understanding structural and functional lateralization. Baboons show human-like lateralized motor behaviors such as handedness for manipulative actions (Molesti et al., 2016; Vauclair et al., 2005) associated with hemispheric specialization of the central sulcus (Margiotoudi et al., 2019), but also patterns of hand preference associated specifically with gestural communication (Meguerditchian & Vauclair, 2006; Meguerditchian et al., 2011). Baboons also have structural brain lateralization in areas homologous to human language areas, including the surface area of the planum temporale in adults (Marie et al., 2018) and newborns (Becker et al., 2021) as well as the Broca’s area homolog (Becker et al., 2022). The latter finding was found to be tied to the manual lateralization of communicative behaviors (Becker et al., 2022), but not non-communicative actions. Thus, the BaBa21 template is well-situated to facilitate volumetric tissue-based analyses in a species that is well-poised to elucidate the evolutionary and developmental origins of language, gesture, and brain lateralization.

There is only one longitudinal developmental template for NHPs currently available, for the cynomolgus macaque (Tan et al., 2025; Zhong et al., 2022). This species has maturational curves for gray and white matter that peak earlier than in humans, with frontal and temporal lobes showing the largest postnatal increase in volume during the first year with a peak of synapse density at 3 months, after which it declines due to pruning (Scott et al., 2016). The timing of baboon cohort scanning was based on macaque maturational trajectories, with the first scan timed to occur prior to the gray and white matter maturation, and the second scan at 9 months to capture development after synaptic pruning, during which time a visible inversion of T2w contrast can be observed (Dehaene-Lambertz & Spelke, 2015). By using both T1w and T2w images in template generation, we took advantage of this additional contrast information for earlier timepoints.

Overall, MRI in nonhuman primates offers numerous advantages for investigating brain development from birth or even prenatal stages. First, most developmental brain MRI databases in humans begin after the age of 4 (e.g., Raznahan et al., 2012), due to the difficulty of obtaining motion-free images at younger ages. By using anesthetized nonhuman primates, motion-free images may be obtained from any age class from birth onward. Related to the first point, the ability to use anesthetized subjects relaxes the time constraints required in human studies, permitting a greater number of MR multimodal sequences and better resolution. Third, longitudinal studies in humans pose significant challenges in terms of recruitment and follow-up, which is why most pediatric neuroimaging studies are cross-sectional (e.g., Jernigan et al., 2016; Van Essen et al., 2013). With NHPs, access to subjects and control of scan timing can be more precisely controlled. Further, NHP colonies themselves permit better control of environmental variables during development, and the opportunity to introduce behavioral experiments and other interventions.

Finally, the interpolation method proposed in this study enables more targeted morphometric analyses or spatial normalization of cohorts of subjects in all age groups. A template can be generated that precisely fits any age within the template timepoint range. This offers advantages for anatomical analyses; for example, voxel-based morphometry approaches, by reducing the bias that could be introduced from a template generated from a less well-matched period in development. This is particularly relevant for studies examining the period between birth and 1 year of age, during which T1w and T2w contrasts undergo significant changes (see Fig. 3).

### 4.2 Limitations and future directions

Following a large cohort of NHP over 5 years for repeated acquisitions presents many challenges. From an initial cohort of 33 individuals, at the time of template building, 21 had reached the age to be scanned at timepoint 3. Going forward, as the cohort matures and all subjects’ scans are acquired for timepoint 3, these will be integrated into a future version of BaBa21. Baboons reach sexual maturity at timepoint 3; however, for thoroughness in the developmental cycle, a timepoint 4 will eventually be added when cohort members reach age 7 and subsequently be integrated into the developmental template.

The BaBa21 template represents the population as a whole and is not divided according to sex. While this template provides a general reference for the species, and can be used to examine sex differences, future work could develop sex-specific templates and investigate potential effects of sexual dimorphism, which might be especially relevant for timepoints during and post adolescence.

In this study, we offer a linear interpolation that estimates the curve of development between timepoints, but we acknowledge that cerebral development is non-linear. Thus, the intermediate templates offered here are estimates that will need to be validated and may be improved in the future as actual scans of individuals at these timepoints. By comparing our intermediate interpolated timepoint templates with an individual scanned at a midpoint between ses-0 and ses-1 (Ozone), we found support for the anatomical accuracy of the volumetric trends, but we acknowledge that our method may not pick up on complex non-linear changes. Nonetheless, these interpolated timepoints are still better targets for registration of scans acquired at intermediate points in development than the original timepoints.

A limitation of the intermediate template generation lies in the interpretation of intensity-based interpolations, particularly in the presence of contrast changes across developmental stages. A “ghosting” effect can be observed in the interpolated images, with the maximum effect potentially occurring between sessions t = 0 and t = 1 at t = 0.5. Further analysis revealed that this effect is not primarily caused by anatomically inconsistent deformations but rather results from contrast inversion in T1-weighted images between consecutive developmental stages. The Supplementary Figure S6 illustrates these intermediate deformations and their associated WM surfaces, showing that the interpolated anatomical structures remain spatially coherent despite the apparent intensity artifacts. Importantly, the figure also demonstrates that this effect is negligible between timepoints with similar contrasts (e.g., t = 1 and t = 2), confirming that the interpolation faithfully preserves anatomical plausibility when image contrast is consistent. These observations indicate that caution is warranted when using an intermediate template (e.g., t = 0.5) for studies focusing on groups with similar age or developmental stage, as intensity-related artifacts may influence analyses in cases of large contrast differences.

Compared to other acquisitions, timepoint 0 required specific processing interventions. The time of acquisition for this timepoint also had some variation, with scans being taken at 2–4 weeks of age. However, this is still well before the known T1/T2 contrast inversion effects take place (Cardoso et al., 2011; Young et al., 2017). White matter segmentation was more challenging, not only due to inversion effects, but also due to the presence of more variable intensities across white matter. This may be due to different rates of myelination occurring during this early window of brain development. On visual inspection, medial white matter appeared to have greater density than distal white matter, suggesting a directional pattern of myelination. This variation, in turn, interfered with intensity-based segmentation. This was addressed in BABACOOL by combining GM and CSF segmentation, and subtracting the binary BM, with some manual corrections. In the future, other approaches might be considered, including incorporating three types of priors for white matter at timepoint 0, corresponding respectively to hyper, hypo, and “regular” signal intensities. This would also facilitate myelination maturation analysis in early development.

### 4.3 Conclusions

BaBa21 offers a normalization target for researchers working with baboon datasets along the majority of brain development, from neonate to sexual maturity. Additionally, the interpolated intermediate template timepoints generated with BABACOOL permits baboon brains of any age up to 5 years to be registered to the template. TPMs further add to the utility of the template, allowing for CSF, WM, and GM separation for volumetric analyses. Over time, the template will be updated to integrate future timepoints with the goal of capturing the full maturational trajectory of *P. anubis*.

We anticipate that this novel resource will contribute to comparative primate development work. To facilitate the use of this resource for standardized analyses, cross-study comparisons, and evolutionary-developmental research in the primate neuroimaging community, we have made the multimodal BaBa21 template available in the PRIMatE Resource Exchange (PRIME-RE; Messinger et al., 2021) website (https://prime-re.github.io/templates_and_atlases_baboon.html#Baba21-a-longitudinal-multimodal-template-of-the-developing-baboon-brain). Comparing baboon, macaque, and human development will permit the elucidation of developmental differences in these species and offer insights into human development and those of model animals used in biomedical research that has implications on human health and cognition.

## Supplementary Material

Supplementary Material

## Data Availability

The full multimodal BABA21 dataset (BIDS formatted) containing all contrasts and TPMs is publicly available on the OpenNeuro platform (Dataset ds005424; Le Troter et al., 2025) at https://openneuro.org/datasets/ds005424. Source code for multimodal (4D+t) BaBa21 template generation and scripts for contrast normalization can be found at https://github.com/arnaudletroter/BABACOOL.
