# Supplementary material for: Longitudinal MRI template of the baboon brain from birth to adolescence
Source: Imaging Neurosci (Camb). 2026 Jul 30;4:IMAG.a.1316. doi: 10.1162/IMAG.a.1316 (PMC13449925; doi:10.1162/IMAG.a.1316)
Supplement: Supplementary file 1 [file IMAG.a.1316_supp.pdf]

Supplementary Information:

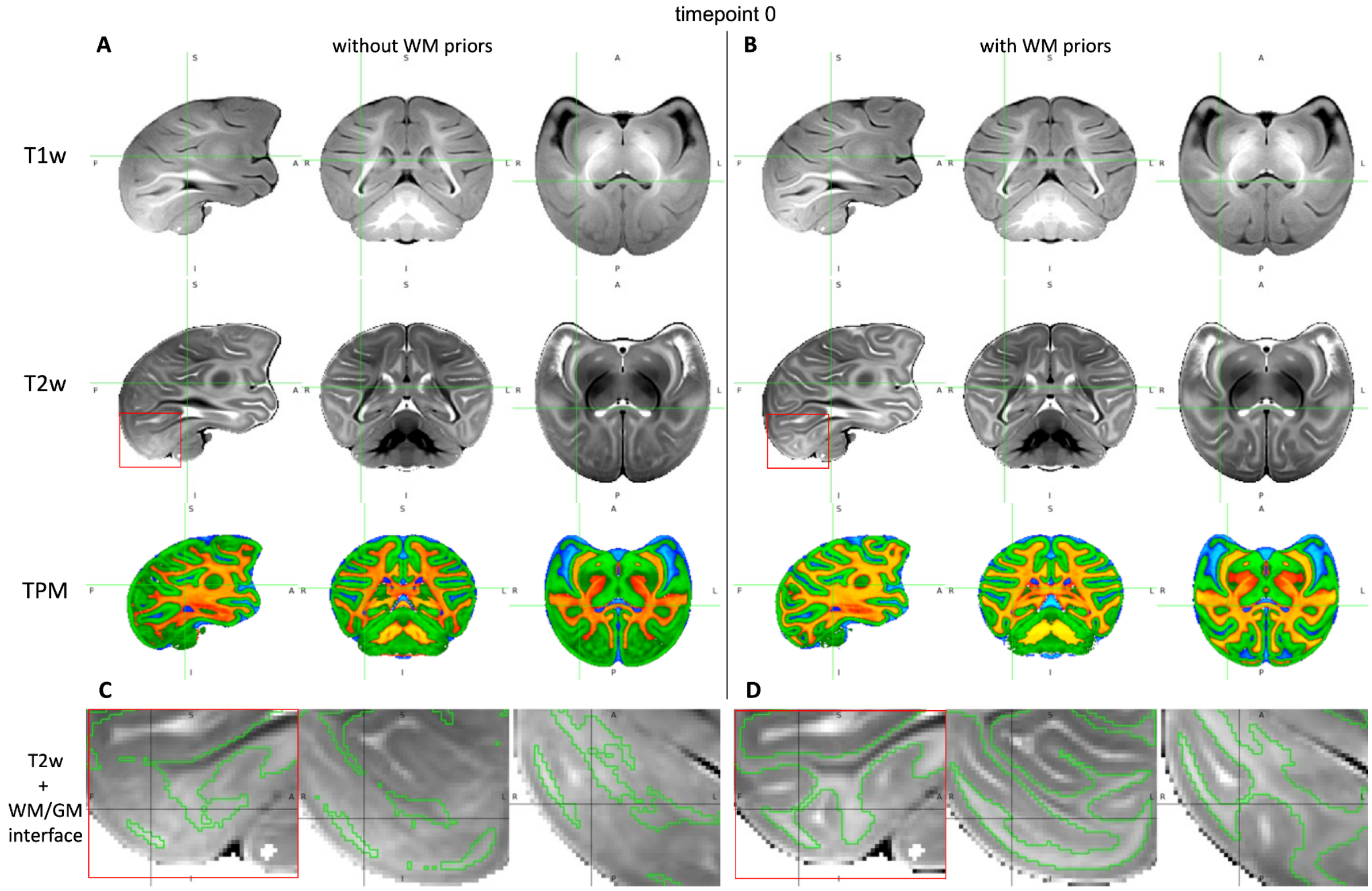

Supplementary Figure 1: Improvement of GM/WM contrasts and segmentation through integration of mask of WM during the SyGN process at timepoint 0. Whole-brain BaBa21 template without WM priors (A) Whole-brain BaBa21 template with WM priors (B) focus on the temporal lobe and WM/GM interface (C) focus on the temporal lobe and WM/GM interface showing better spatial coherence (D)

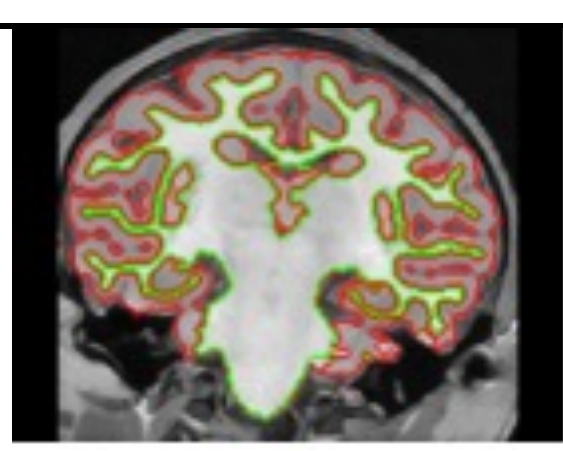	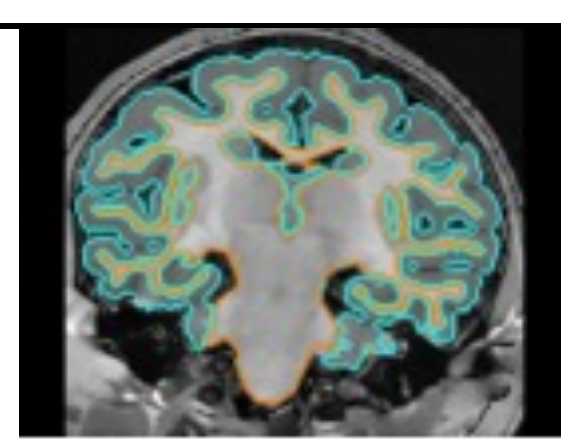
**Ozo-1 segmentation**<br>vol(WM)= 35676 (green)	**ozo-1 segmentation warped onto ozo-0**<br>vol(WM)= 33971 (orange)

Supplementary Figure 2: Estimated WM volume change between ozo-0 and ozo-1.

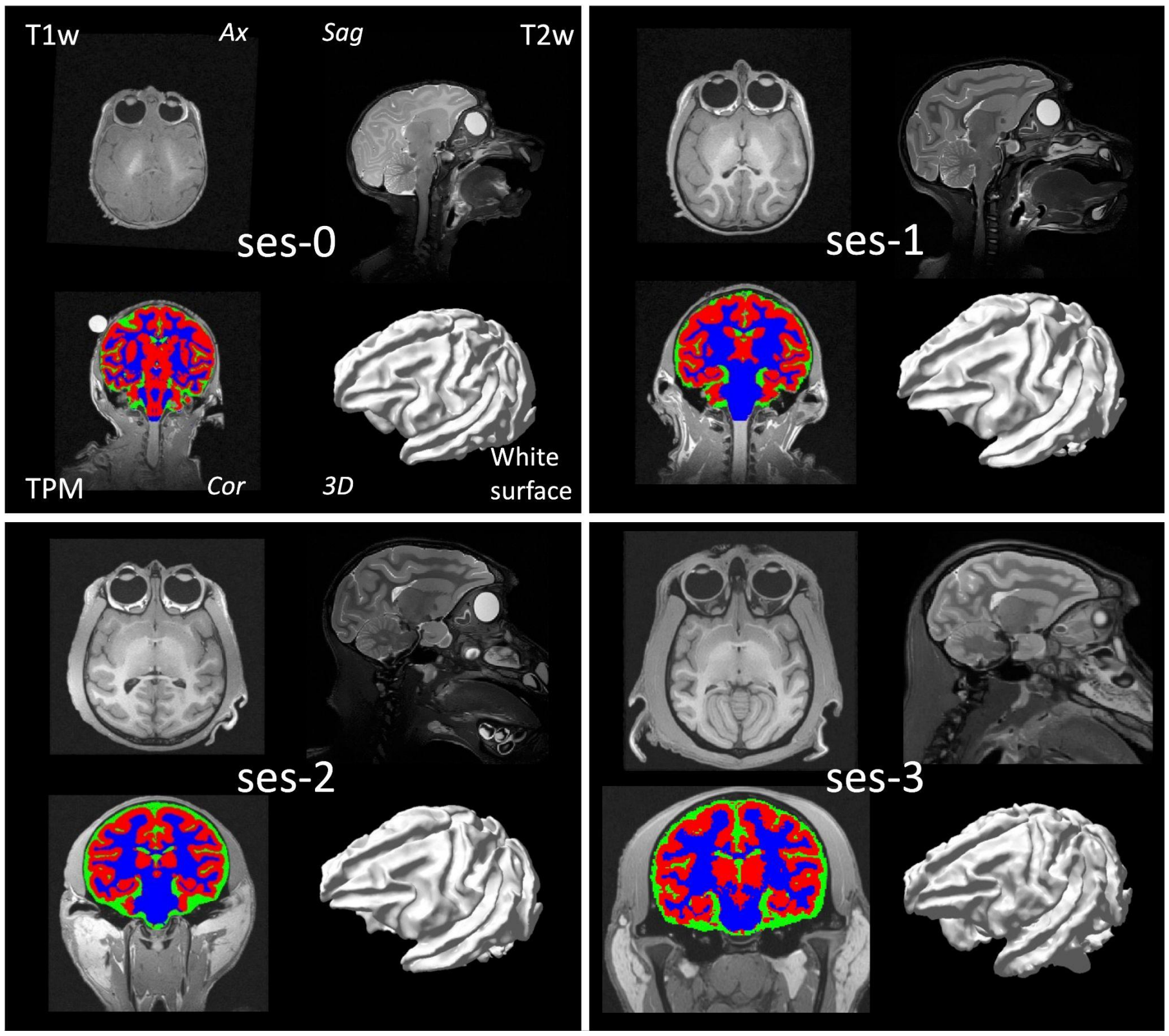

Supplementary Figure 3: Examples of manual segmentation for a single individual (Prune) across all timepoints; gray matter (green), white matter (red), and CSF (blue).

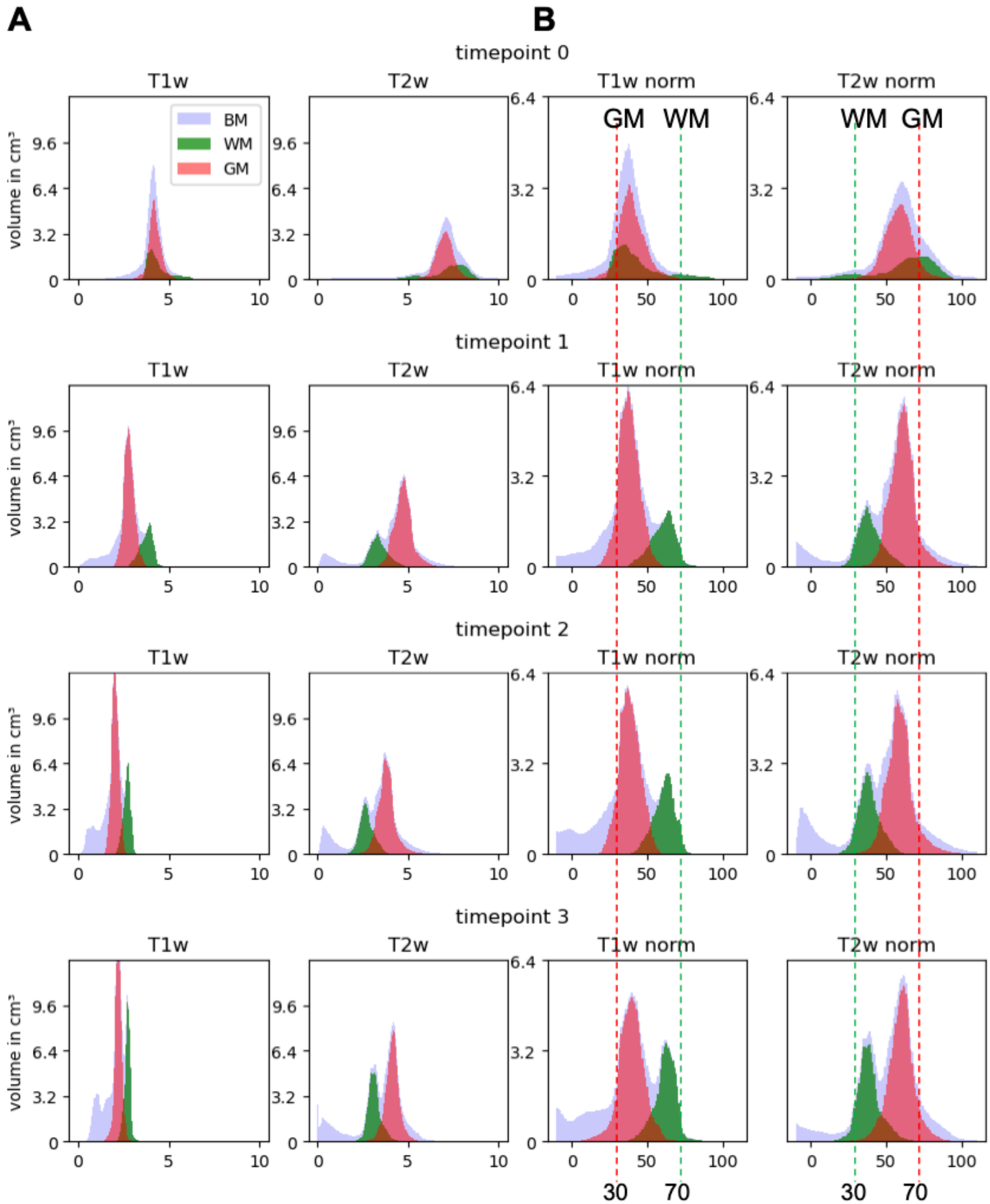

Supplementary Figure 4: Results of TPM histogram-based normalization for T1w and T2w, across all timepoints, before normalization on left (A), after normalization on right (B).

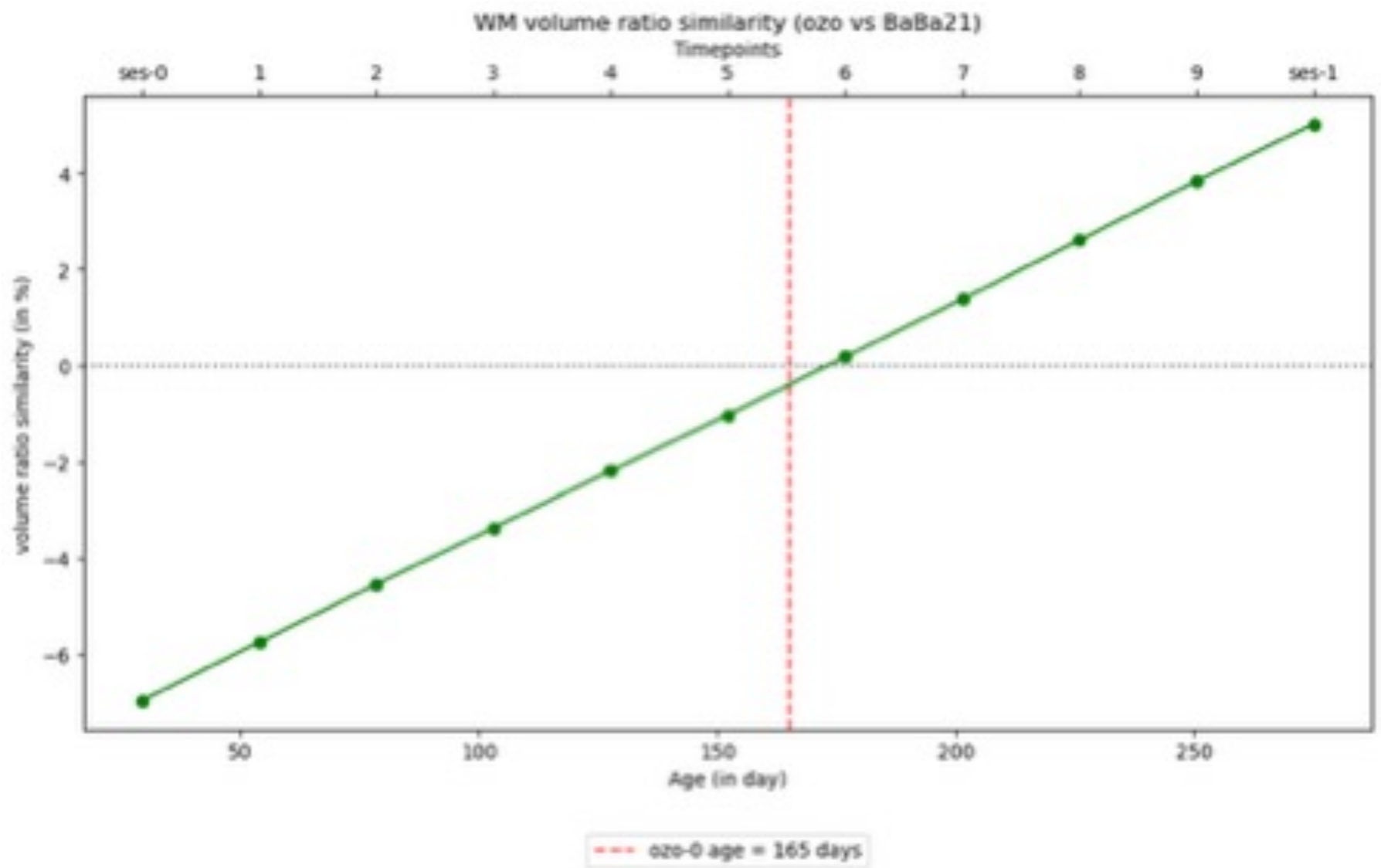

Supplementary Figure 5: Volume ratio similarity between ozo-0 WM volumes (175 days) and intermediate BaBa21 timepoint 6, which corresponds to approximately 175 days.

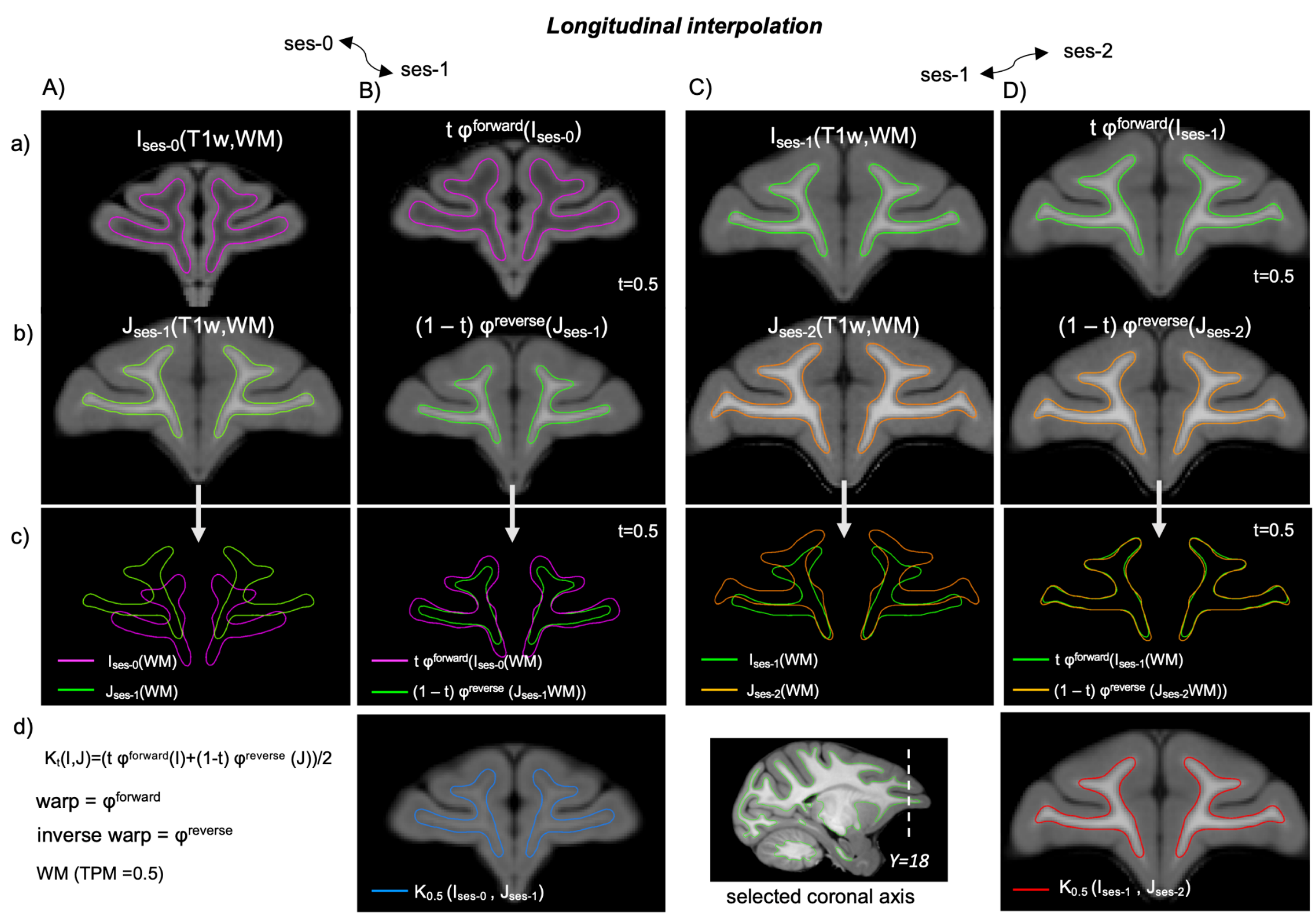

Supplementary Figure 6: intermediate deformations between developmental sessions. Columns A and B correspond to interpolations between sessions ***ses-0 and ses-1***, while columns C and D correspond to interpolations between sessions ***ses-1 and ses-2***.

Row a) shows, in the coronal slice (Y = 18), the T1w volume and white matter (WM) surface of the source volume $I_{ses\text{-}0}$ along with the forward deformation $\varphi^{forward}$ weighted by (t = 0.5).

Row b) displays the target T1w and WM images $J_{ses\text{-}1}$ and the corresponding inverse deformation $\varphi^{reverse}$ (t=0.5)

Row c) presents the superposition of WM surfaces before registration (column A) and after registration and interpolation (column B)

Row d) shows the results of the interpolation, with WM surfaces in blue highlighting at the intermediate timepoint (t=0.5)

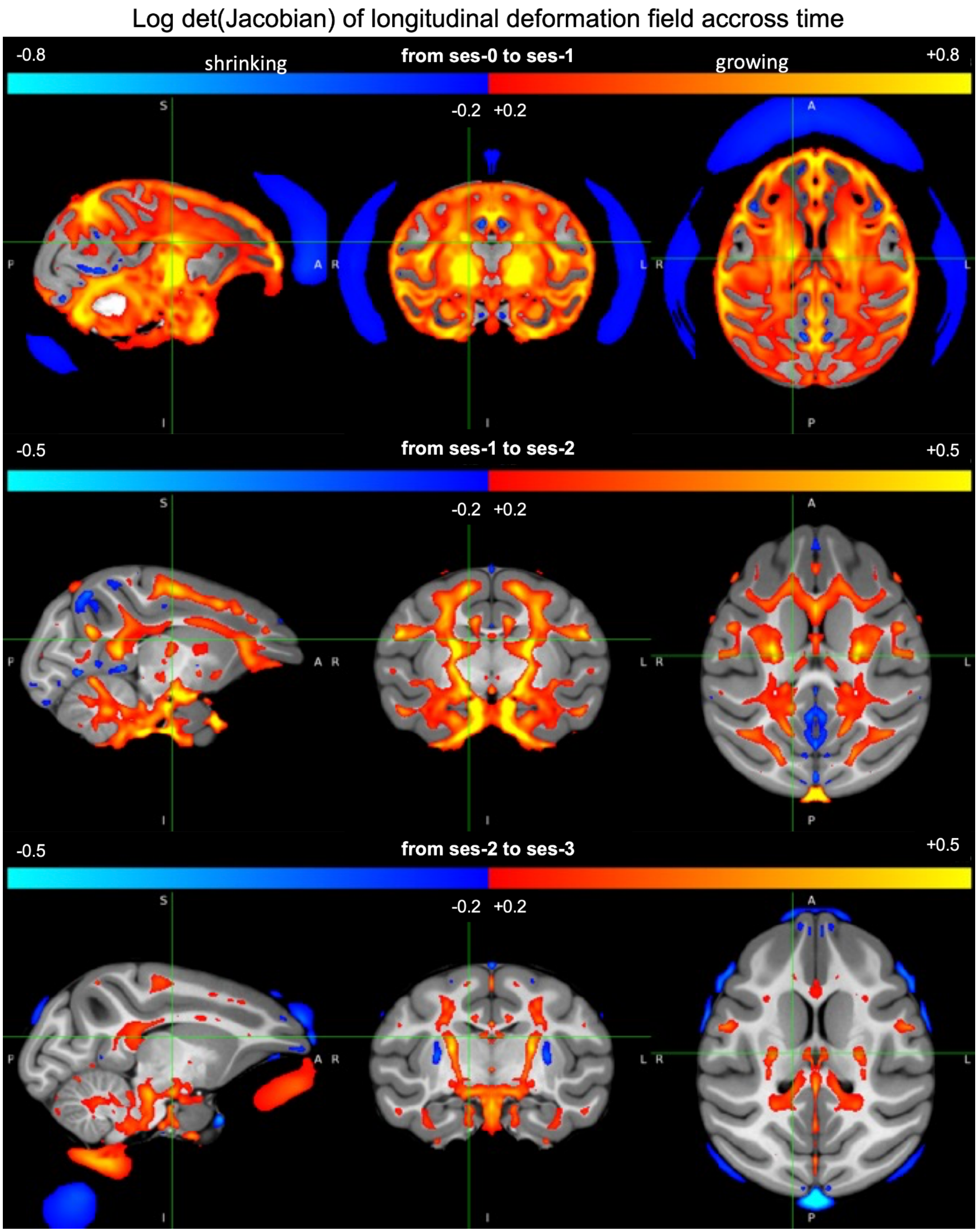

Supplementary Figure 7: Log Jacobian determinant (log|J|) for the full forward deformation fields between successive timepoints.
